# Developing a 5-gene prognostic signature for cervical cancer by integrating mRNA and copy number variations

**DOI:** 10.1186/s12885-022-09291-z

**Published:** 2022-02-21

**Authors:** Wenxin Liu, Qiuying Jiang, Chao Sun, ShiHao Liu, Zhikun Zhao, Dongfang Wu

**Affiliations:** 1 Department of Gynecological Oncology, Tianjin Medical Cancer Institute and Hospital, National Clinical Research Center for Cancer, Key Laboratory of Cancer Prevention and Therapy,Tianjin, Tianjin’s Clinical Research Center for Cancer, West Huan-Hu Rd, Ti Yuan Bei, Hexi District, 300060 Tianjin, China; 2 Department of Internal Medicine, Second Affiliated College of Harbin Medical University, 246 Xuefu Road, Nangang District, Harbin City, 230100 Heilongjiang Province China; 3 YuceBio Technology Co., Ltd, 4th floor, phase I, dabaihui center, no.2002, Shenyan Road, Haishan street, Yantian District, Shenzhen City, 440300 Guangdong Province China; 4 Department of Obstetrics and Gynecology, The Fourth Hospital of Hebei Medical University, NO.12, JianKang Road, Shijiazhuang, 130100 Hebei Province China

**Keywords:** Cervical cancer, Copy number variations, Differential expressed genes, Prognostic signature, Bioinformatics

## Abstract

**Background:**

Cervical cancer is frequently detected gynecological cancer all over the world. This study was designed to develop a prognostic signature for an effective prediction of cervical cancer prognosis.

**Methods:**

Differentially expressed genes (DEGs) were identified based on copy number variation (CNV) data and expression profiles from different databases. A prognostic model was constructed and further optimized by stepwise Akaike information criterion (stepAIC). The model was then evaluated in three groups (training group, test group and validation group). Functional analysis and immune analysis were used to assess the difference between high-risk and low-risk groups.

**Results:**

The study developed a 5-gene prognostic model that could accurately classify cervical cancer samples into high-risk and low-risk groups with distinctly different prognosis. Low-risk group exhibited more favorable prognosis and higher immune infiltration than high-risk group. Both univariate and multivariate Cox regression analysis showed that the risk score was an independent risk factor for cervical cancer.

**Conclusions:**

The 5-gene prognostic signature could serve as a predictor for identifying high-risk cervical cancer patients, and provided potential direction for studying the mechanism or drug targets of cervical cancer. The integrated analysis of CNV and mRNA expanded a new perspective for exploring prognostic signatures in cervical cancer.

**Supplementary Information:**

The online version contains supplementary material available at 10.1186/s12885-022-09291-z.

## Background

Human papillomavirus (HPV) vaccine uptake could prevent the incidence of cervical cancer, but according to global cancer statistics, in 2020 there were 13.3 per 100 000 women suffering from cervical cancer, and 604 127 new cases were diagnosed [1]. Surgery and chemotherapy are the main strategies for treating cervical cancer, and the International Federation of Gynecology and Obstetrics (FIGO) has also developed a staging system for personalized therapy [2]. Patients classified as having a low risk recurrence by FIGO can still develop metastasis [3], which will inevitably increase the difficulties of treatment. Therefore, discovery of efficient predictor may help predict the prognosis and guide personalized treatment of cervical cancer.

In the recent years, various biomarkers, such as immune genes [4], long non-coding RNAs [5, 6], microRNAs [7, 8] and histone genes [9], have been discovered to evaluate the prognosis of cervical cancer patients. Up to now, prognostic signature based on copy number variations (CNVs) has not been investigated before. A number of studies have demonstrated that CNVs are involved in tumorgenesis in many cancer types, such as lung cancer [10], leukaemia [11] and breast cancer [12]. In a pan-cancer research, Shao et al. revealed a close relation between CNVs and gene expression enriched in oncogenic pathways [13]. Advances in gene microarray technology enable us to detect duplications or deletions from focal to chromosomal associated with cancer development using various databases.

Tumor microenvironment (TME) plays a critical role in cancer cell proliferation, metastasis and immune escape. Particularly, to a large extent, the efficacy of immunotherapy is determined by TME [14]. Immunotherapy is a potentially effective strategy for cervical cancer patients with metastasis. Immune checkpoint blockade such as programmed death receptor-1 (PD-1) and CTLA-4 inhibitors has been seen as re-activators for T cell activation [15]. Currently, there are ongoing clinical trials exploring immune checkpoint inhibitors for aggressive cervical cancer.

In this study, cervical cancer samples were obtained from The Cancer Genome Atlas (TCGA) and Gene Expression Omnibus (GEO) databases, where TCGA and GSE44001 datasets containing expression data and sequencing data were downloaded. We constructed a prognostic signature for cervical cancer patients according to combined data of CNVs and mRNAs. The effectiveness of the prognostic signature was validated using TCGA and GSE44001 datasets. The signature was robust in dividing patients into high-risk and low-risk groups, which showed distinctly different overall survival (OS). A nomogram was proposed based on the prognostic signature to satisfy a convenient clinical use. In addition, TME of high-risk and low-risk groups was described for understanding immune infiltration in the two groups. The current prognostic signature manifested a robust performance through comparison with previously reported signatures.

## Methods

### Data source

**Fig. 1:**
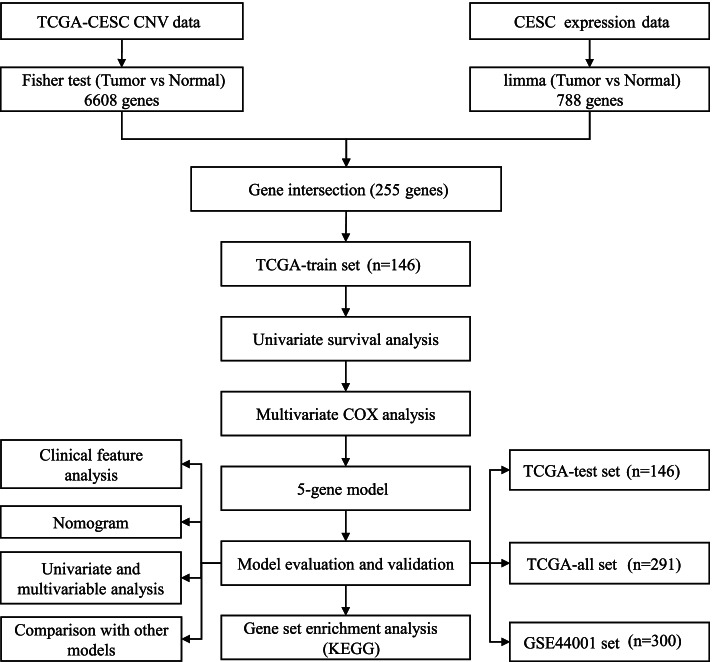
The workflow of constructing a prognostic model for cervical cancer

The workflow of constructing a prognostic model for cervical cancer was shown in Fig. 1. The data of cervical cancer samples were downloaded from TCGA (https://portal.gdc.cancer.gov/) database and GEO (https://www.ncbi.nlm.nih.gov/geo/) database on August 30, 2021. TCGA dataset included RNA sequencing (RNA-seq) data, CNV data and clinical information. GSE44001 from GEO included expression profiles. 10 normal samples of cervix uteri containing expression profiles were downloaded from GTEx database (https://www.gtexportal.org/) on August 30, 2021.

### Data preprocessing

For TCGA dataset, samples without clinical information, survival status (dead and alive) or survival time were excluded. Primary solid tumor and normal solid tissue were retained. “RemoveBatchEffect” function in limma R package [16] was used to remove batch effects between TCGA and GTEx datasets (Supplementary Figure S1). For GSE44001 dataset, samples without survival status were excluded. Probe ID was converted to gene symbol. One probe containing multiple genes was excluded. When one gene had multiple probes, averaged expression value of these probes was selected. Finally, 291 samples were remained in TCGA dataset, which were grouped by survival status (220 alive and 71 dead), T stage (T1-137, T2-67, T3-16, T4-10, TX-61), N stage (N0-128, N1-55, NX-108), M stage (M0-107, M1-10, MX-174), stage (I-159, III -64, III-41, IV-21, X-6), grade (G1-18, G2-129, G3-116, G4-1, GX-27), age (139 samples ≤ 45 years and 152 samples > 45 years) and HPV status (positive-9, negative-167, NA-115). In GSE44001 dataset, 300 samples were remained with 262 alive and 38 dead samples. The clinical information of these samples was displayed (Supplementary Table S1).

### Identification of differentially expressed genes (DEGs)

DEGs were obtained from differential CNVs and differential genes. For CNV data, bedtoolsr R package [17] was applied to transform CNV segments to genes, and CNVs in tumor samples and normal samples were calculated. The differential CNVs were identified by *Chi*-square test with *P* < 0.05. For expression profiles, limma R package was used to identify DEGs between normal samples in GTEx dataset and tumor samples in TCGA dataset (*P* < 0.01, |fold change (FC)|> 1.5). Finally, the intersection of genes in differential CNVs and DEGs in the expression profiles was the gene set of DEGs.

### Analysis of GO function and KEGG pathways

WebGestaltR package [18] was applied to analyze Gene Ontology (GO) function and Kyoto Encyclopedia of Genes and Genomes (KEGG) pathways. WebGestaltR is a popular tool supporting various functional categories and databases when performing enrichment analysis. GO function includes molecular function, cellular component and biological process. Only the top 10 enriched terms or pathways were visualized.

### Construction of a prognostic model

TCGA dataset was randomly divided into training group and test group at a ratio of 1:1. The suitable division was selected under the conditions of similar distribution of two groups in ages, genders, survival status, follow-up time and similar number of samples of binary classification for expression profiles. Finally, 146 samples in training group and 145 samples in test group were determined. *Chi*-square test showed no significant difference between two groups (*P* > 0.05, Supplementary Table S2). Moreover, on the univariate Cox regression analysis of genes in training and test groups, a similar distribution of their *P* values was displayed, indicating that the classification of two groups was reliable (Supplementary Figure S2). GSE44001 was set as an independent validation group.

Survival coxph function in survival R package was used to perform univariate Cox regression analysis in training group, and *P* < 0.05 was set to screen DEGs. StepAIC in MASS package [19] was applied for model optimization. The AIC of the model decreased by decreasing variables one by one, with a lower AIC reflecting a more optimized model. The prognostic model was defined as risk score = coefficient 1* gene expression 1 + coefficient 2* gene expression 2 + … + coefficient n* gene expression n, where coefficients were obtained from the result of univariate Cox regression analysis. Risk score was converted to z-score to classify samples into high-risk and low-risk groups through z-score = 0. TimeROC R package [20] was used to show receiver operating characteristic (ROC) curves, and area under ROC curve (AUC) was calculated to evaluate the prediction of the prognostic model. Kaplan–Meier survival analysis was employed to analyze the survival, the differences of which in the two risk group were analyzed by log-rank test.

### Estimation of STromal and Immune cells in MAlignant Tumours using Expression data (ESTIMATE)

ESTIMATE is a method that can evaluate the fraction of stromal and immune cells based on gene expression signatures through single sample gene set enrichment analysis (ssGSEA) [21]. The method calculates three enrichment scores, that is, stromal score, immune score and ESTIMATE score, where ESTIMATE score is the combined score of stromal score and immune score.

### Microenvironment Cell Populations-counter (MCP-counter)

MCP-counter calculates the enrichment score of 10 immune-related cells (CD3 T cells, CD8 T cells, cytotoxic lymphocytes, B lymphocytes, NK cells, monocytic lineage, myeloid dendritic cells, neutrophils, endothelial cells and fibroblasts) across mRNA mixtures in tumor tissue [22]. This tool enables to estimate relative abundance of immune-related cells based on a series of cell markers in a complex tumor microenvironment.

### Single sample gene set enrichment analysis

GSVA R package was used to conduct ssGSEA for determining enrichment score of a gene set in one sample [23]. With this method, the abundance of gene expression can be calculated and compared between different groups. TME was analyzed using ssGSEA to obtain the enrichment of 28 immune cells.

### Construction of a nomogram

Visualization of a nomogram allows a direct prediction of overall survival based on a series of risk factors. We included the risk factors with hazard ratio (HR) > 1 (*P* < 0.05) from multivariate Cox regression analysis. Each risk factor was assigned with a score, and total points of risk factors corresponded to survival chance of 1-year, 3-year and 5-year period.

### Decision curve analysis (DCA)

To objectively compare the different factors in survival prediction, DCA, which enables a standard comparison for evaluating performance of predictive factors in clinical decision based on net benefit, was introduced here to evaluate the cost performance of nomogram, risk score and other clinical features. The methodology is commonly used in evaluating predictive models for clinical use [24, 25].

### Statistical analysis

R (version 3.4.2) software was used to conduct all statistical analysis and bioinformatics analysis. *P* < 0.05 was considered as significant. Statistical methods were presented in the corresponding figure legends. All parameters were defined as default if there was no specific descriptions.

## Results

### Identification of DEGs based on CNV data and expression profiles

**Fig. 2:**
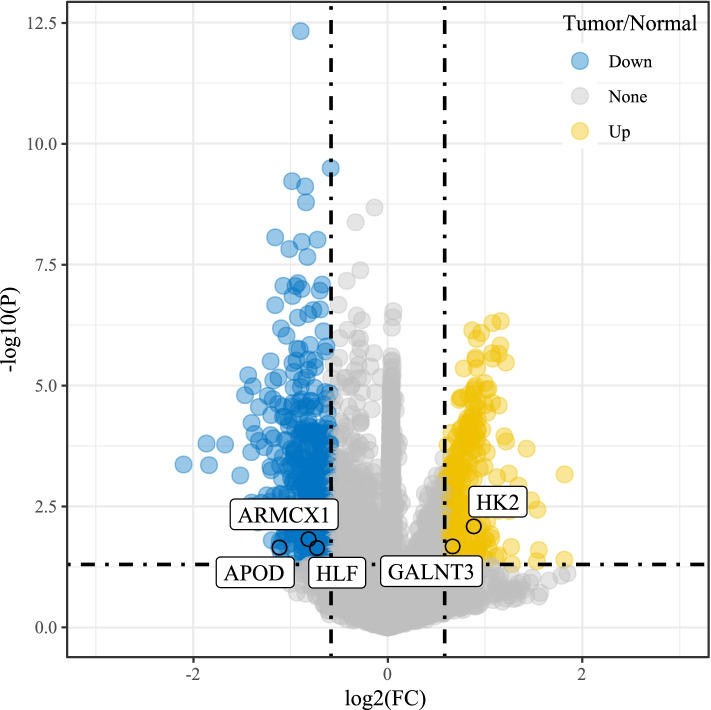
A volcano plot of up-regulated (yellow) and down-regulated (blue) genes identified from expression profiles. The vertical dotted line indicates |FC|= 1, and the horizontal dotted line indicates *P* = 0.01. FC, fold change

**Fig. 3:**
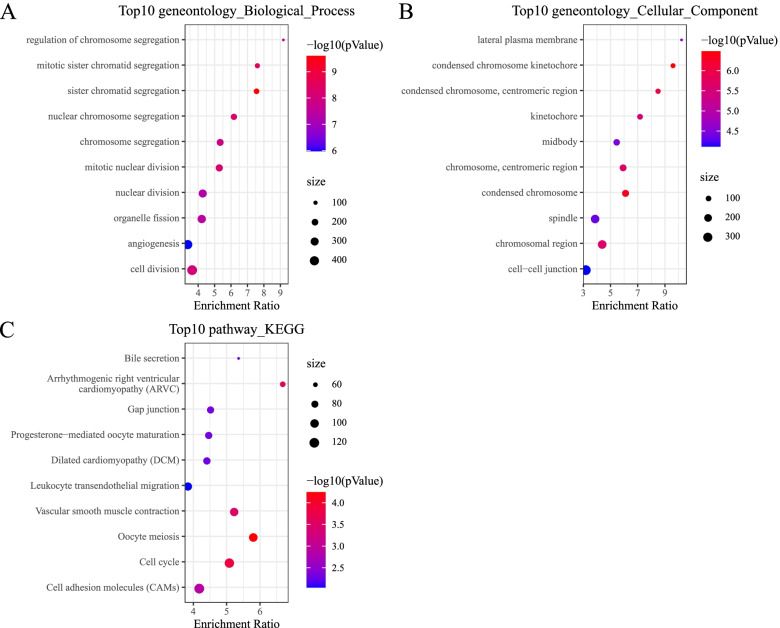
Analysis of GO function and KEGG pathways. **A-C** The top 10 enriched terms of biological process **A**, cellular component **B** and KEGG pathways **C**. *P* value was presented as – log10 (*P* value). Dot size represents the number of genes enriched in one term

For CNV data in TCGA dataset, we analyzed the CNV of each sample by comparing with normal samples, and 6608 differential CNVs containing 6608 genes were screened (*P* < 0.05). 788 DEGs including 268 up-regulated and 520 down-regulated genes were identified (*P* < 0.01 and |FC|> 1.5, Fig. 2). A Venn diagram was plotted for discovering the common genes between 6608 genes from differential CNVs and 788 DEGs (Supplementary Figure S3). Functional analysis on 255 intersected DEGs annotated 282 terms of biological processes and 86 terms of cellular components (*P* < 0.05, Fig. 3A and B, Supplementary Table S3), but no molecular function was significantly enriched. We found that cell cycle-related terms, such as chromosome segregation and nuclear division, were significantly enriched. In addition, 12 KEGG pathways were significantly annotated, and the top 10 enriched pathways were shown (*P* < 0.05, Fig. 3C). Several pathways related to cell signaling and cell proliferation, such as cell cycle, gap junction, leukocyte transendothelial migration and cell adhesion molecules, were enriched.

### Construction of a prognostic model based on 255 DEGs

Based on 255 DEGs identified from CNV data and mRNA data, we attempted to develop a prognostic model for cervical cancer. Univariate Cox regression analysis was conducted to detect genes significantly associated with prognosis. Totally 11 genes were screened (Supplementary Table S4), and then stepAIC was used to reduce the number of genes for constructing an optimal model. Finally, 5 genes (*APOD*, *ARMCX1*, *GALNT3*, *HK2* and *HLF*) remained, and the prognostic model was defined as follow:

Risk score =—0.238*APOD + 0.462*ARMCX1 + 0.503*GALNT3 + 0.406*HK2 -0.407*HLF.

The distribution of these 5 genes were reflected in a genome map, with *APOD* in chromosome 3, *ARMCX1* in chromosome X, *GALNT3* and *HK2* in chromosome 2, and *HLF* in chromosome 17 (Supplementary Figure S4). The expression of the 5 genes in TCGA samples was significantly changed compared with normal samples, specifically, *APOD*, *ARMCX1* and *HLF* were down-regulated, and *GALNT3* and *HK2* were up-regulated (Supplementary Figure S5). We also found an obvious correlation between the expression of three genes (*ARMCX1*, *GALNT3* and *HK2*) and CNV (Supplementary Figure S6).

### Evaluation of the 5-gene prognostic model

**Fig. 4:**
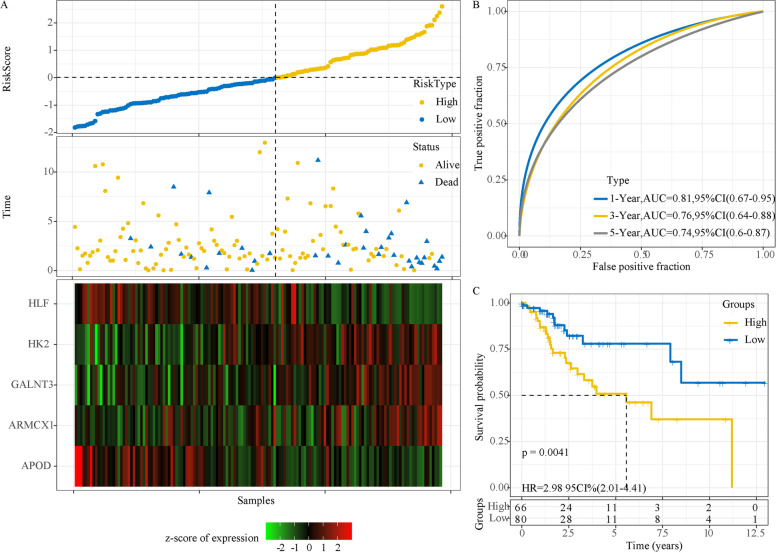
The performance of the 5-gene prognostic model in training group. **A** The survival status and expression of 5 genes of each sample ranked by risk score. The expression level was converted to z-score. Red indicates z-score > 0 and relatively high expression, while green indicates z-score < 0 and relatively low expression. B ROC curve of predicting 1-year, 3-year and 5-year survival. **C** Kaplan–Meier survival curve of high-risk and low-risk groups. Log-rank test was performed. AUC, area under ROC curve. CI, confidence interval. HR, hazard ratio

Firstly, we randomly divided 291 samples from TCGA dataset into two groups, with 146 samples as a training group and 145 samples as a test group (Supplementary Table S2). The risk score for each sample was calculated according to the expression of 5 prognostic genes. Risk score was converted to z-score, and z-score = 0 was set as a cut-off to classify samples into high-risk and low-risk groups in the training group (Fig. 4A). It was observed that samples of dead status were more enriched in high-risk group, and that the expression level of 5 genes were significantly distinct between two risk groups. ROC analysis revealed a high AUC of 1-year (0.81), 3-year (0.76) and 5-year (0.74) OS prediction (Fig. 4B), indicating the effectiveness of the prognostic model. Kaplan–Meier survival analysis also showed a significant classification of high-risk group with 66 samples and low-risk group with 80 samples (*P* = 0.0041, HR = 2.98, 95%CI = 2.01–4.41, Fig. 4C).

**Fig. 5:**
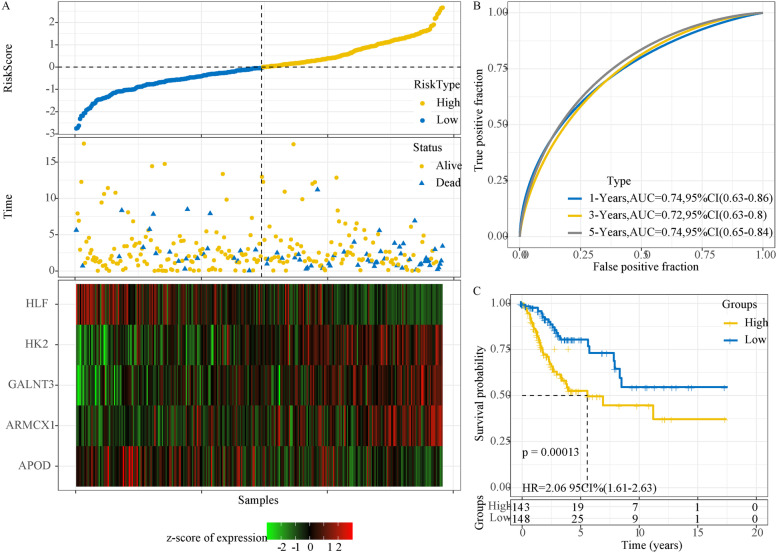
The performance of the 5-gene prognostic model in TCGA dataset. **A** The survival status and expression of 5 genes of each sample ranked by risk score. The expression level was converted to z-score. Red indicates z-score > 0 and relatively high expression, while green indicates z-score < 0 and relatively low expression. **B** ROC curve of 1-year, 3-year and 5-year survival predicting. **C** Kaplan–Meier survival curve of high-risk and low-risk groups. Log-rank test was performed. AUC, area under ROC curve. CI, confidence interval. HR, hazard ratio

The prediction of the prognostic model was further evaluated in the test group, and the model manifested the similar results when compared with the training group (Supplementary Figure S7). 145 samples were neatly classified into high-risk and low-risk groups, with a favorable AUC of 1-year (0.69), 3-year (0.68) and 5-year (0.76). Survival analysis showed that the risk score could classify patients into two groups with distinct OS (*P* = 0.0044, Supplementary Figure S7C). The distribution of risk score for total samples in TCGA dataset was shown (Fig. 5). 143 samples and 148 samples were classified into high-risk and low-risk groups, respectively, with differential OS (*P* = 0.00013, HR = 2.06, 95%CI = 1.61–2.63, Fig. 5C). Furthermore, we included an independent dataset (GSE44001) to validate the robustness of the 5-gene prognostic model, and obtained similar results that the samples were significantly divided into high-risk and low-risk groups with distinctly different prognosis (*P* = 0.0059, Supplementary Figure S8). Therefore, the 5-gene prognostic model was effective in distinguishing high-risk and low-risk for cervical cancer patients.

### Risk score is associated with clinical features

To assess the relation between risk score and clinical features, we evaluated the distribution of risk score in different clinical features (Supplementary Figure S9). There was a higher proportion of alive samples in low-risk group (*P* < 0.05, Supplementary Figure S9A). Although no significant difference was observed among different stages, a higher proportion of samples was observed in advanced cancer stages (Supplementary Figure S9B-E). In different ages, genders and HPV status, there was no differential distribution of risk score (Supplementary Figure S9F and G).

**Fig. 6:**
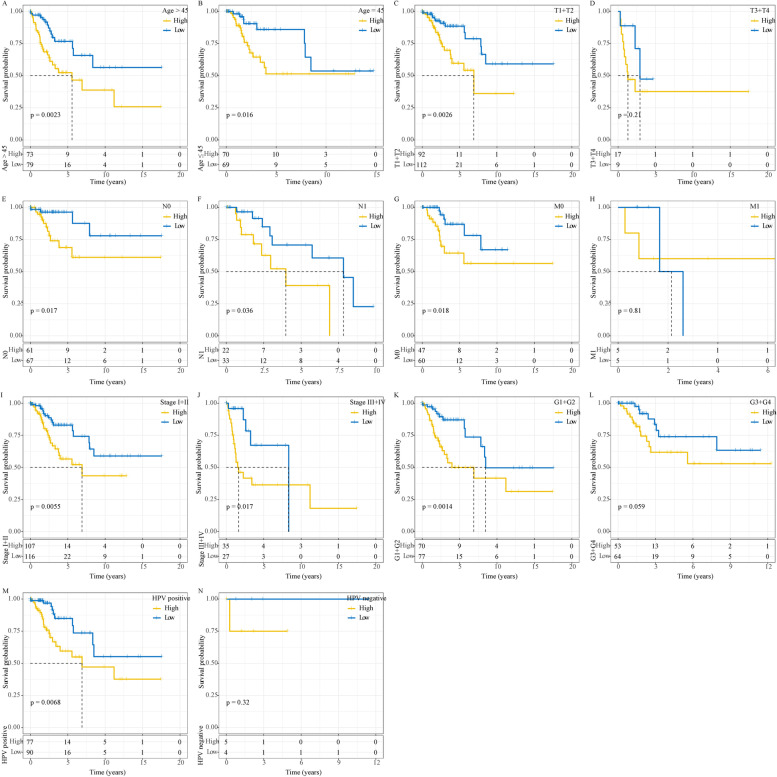
Kaplan–Meier survival curves of high-risk and low-risk groups in different clinical features including ages **A-B**, T stage **C-D**, N stage **E–F**, M stage **G-H**, stage **I-J**, grade **K-L** and HPV status **M–N** in TCGA dataset. Log-rank test was performed. Blue represents low-risk group and yellow represents high-risk group. Log-rank test was performed

Moreover, we analyzed if risk score could classify patients into high-risk and low-risk groups with different clinical features. The results manifested that risk score was also robust in sample classification into high-risk and low-risk groups in different clinical features, including age > 45 and age ≤ 45, T1 and T2 stages, N0 and N1 stages, M0 stage, stage I and II, stage III and IV, grade 1 and 2, grade 3 and 4, and HPV-positive (*P* < 0.05, Fig. 6).

**Fig. 7:**
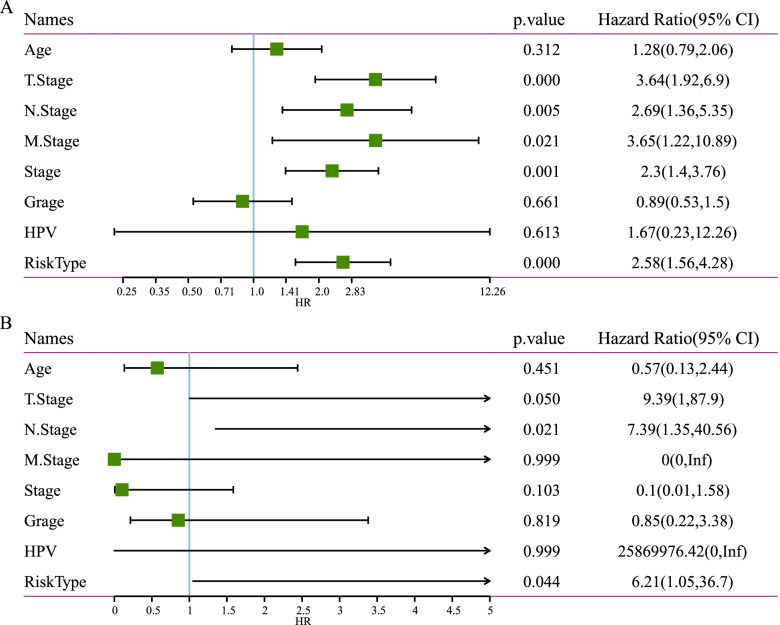
Univariate **A** and multivariate **B** Cox regression analysis of clinical features and risk score in TCGA dataset. Log-rank test was performed. CI, confidence interval. HR, hazard ratio

To assess the independence of the 5-gene prognostic signature in clinical use, we applied univariate and multivariate Cox regression analysis on TCGA dataset. Univariate Cox regression analysis revealed that T stage, N stage, M stage, stage and risk type were the risk factors for cervical cancer patients (*P* < 0.05, Fig. 7A). From the data of multivariate Cox regression analysis, T stage, N stage and risk score were considered as risk factors (Fig. 7B). High HR of risk type was presented in the univariate and multivariate Cox regression analysis, with HR = 2.58 (*P* < 0.0001, 95%CI = 1.56–4.28) and HR = 6.21 (*P* = 0.044, 95%CI = 1.05–36.7), respectively.

### Constructing a nomogram based on risk score

**Fig. 8:**
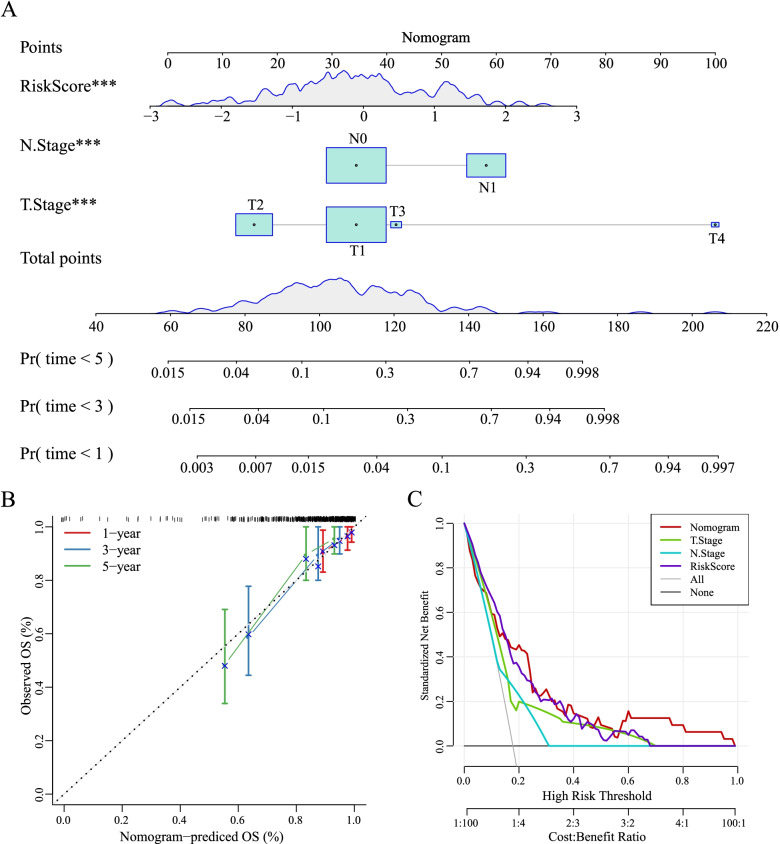
A nomogram based on risk score and TCGA dataset for clinical use. **A** A nomogram based on risk score, N stage and T stage. 1-year, 3-year and 5-year OS was predicted as death rate. **B** Correction of predicted OS in 1-year, 3-year and 5-year period. **C** DCA curve of nomogram, risk score, T stage and N stage. Vertical axis represents the net benefit of before and after receiving treatment. Grey line indicates that all samples were positive and received treatment. Black line indicates that all samples were negative and received no management

To satisfy a convenient clinical use, we developed a nomogram that can directly exhibit the prognostic model to predict prognosis. Three risk factors (T stage, N stage and risk score) selected based on the results of multivariate Cox regression analysis and TCGA samples were included to construct a nomogram (Fig. 8A). Each risk factor can obtain a score, and total points indicates the death rate of 1-year, 3-year and 5-year survival. Predicted OS was corrected then by the observed OS (Fig. 8B). Furthermore, DCA was used to evaluate the efficiency of the nomogram, and we found that the nomogram could more accurately assist clinical decision than other predictors in prognosis prediction (Fig. 8C).

### Differential immune features between high-risk and low-risk groups

**Fig. 9:**
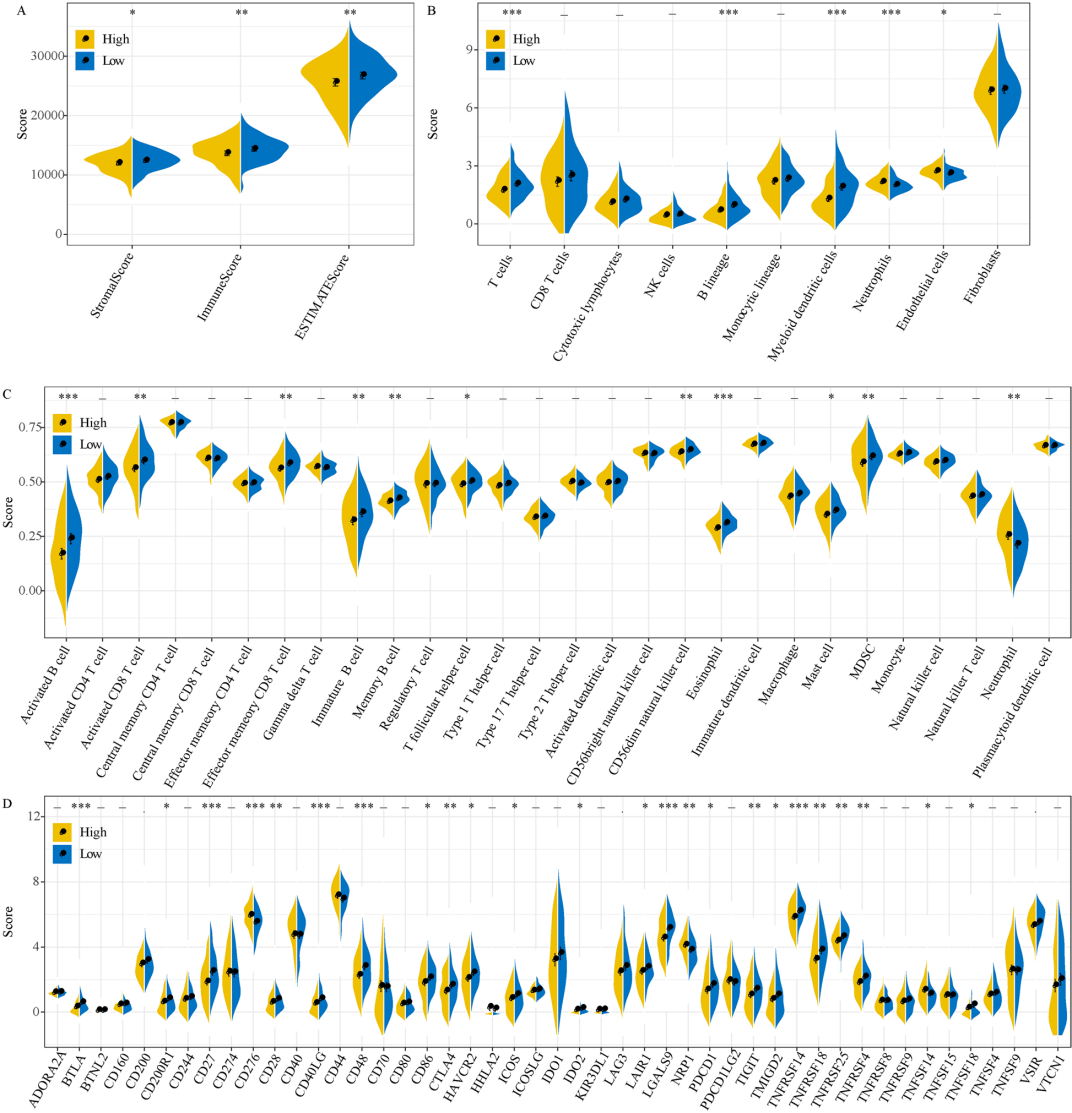
The relation between risk score and tumor immune microenvironment analyzed in TCGA dataset. **A** ESTIMATE score, stromal score and immune score analyzed by ESTIMATE measurement. **B** Enrichment score of 10 immune cells calculated by MCP-counter. **C** Enrichment score of 28 immune cells calculated by ssGSEA. **D** Enrichment score of 47 immune checkpoints in high-risk and low-risk groups. Student *t* test was performed. *P < 0.05, **P < 0.01, ***P < 0.001

Tumor microenvironment plays an important role in cancer development and immune escape, therefore we analyzed the relation between immune infiltration and risk score through ESTIMATE, MCP-counter and ssGSEA. ESTIMATE revealed that low-risk group had higher stromal score and immune score than high-risk group, indicating high immune infiltration in low-risk group (*P* < 0.01, Fig. 9A). The MCP-counter results showed that T cells, B lineage and myeloid dendritic cells contributed to a higher enrichment in low-risk group, while neutrophils and endothelial cells were higher-enriched in high-risk group (*P* < 0.05, Fig. 9B). Additionally, we evaluated 28 types of immune cells obtained from a previous study through ssGSEA [26]. Activated B cells, immature B cells, activated CD8 T cells, effector memory CD8 T cells and myeloid-derived suppressor cells (MDSC) had significantly high enrichment score in low-risk groups (*P* < 0.05, Fig. 9C), which suggested a more activated immune response in low-risk group than high-risk group. Moreover, we assessed the expression of 47 immune checkpoints obtained from Danilova et al. [27]. 24 of 47 immune checkpoints exhibited differential expression between high-risk and low-risk groups (*P* < 0.05, Fig. 9D), meaning that the differential expression of these immune checkpoints may result in differential immune response.

### Functional pathways related to risk score

**Fig. 10:**
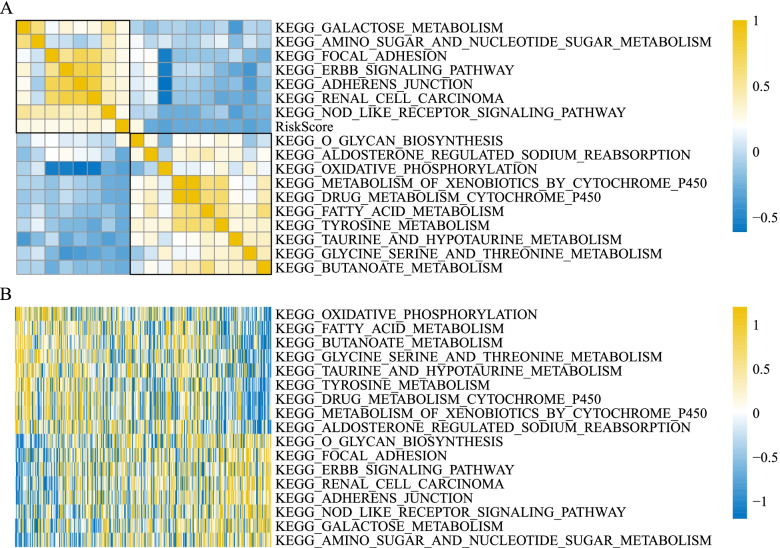
KEGG pathways significantly associated with risk score. **A** Pearson correlation analysis between risk score and KEGG pathways with correlation coefficient > 0.25. Yellow represents positive correlation and blue represents negative correlation. *Chi*-square test was performed. **B** Enrichment of 17 KEGG pathways significantly associated with risk score. Yellow represents relatively high enrichment score and blue represents relatively low enrichment score

To evaluate the relation between risk score and enrichment of functional pathways, we used ssGSEA to calculate the enrichment score of KEGG pathways for each sample in TCGA dataset. Pearson correlation analysis revealed that 17 KEGG pathways were enriched and correlated with risk score (*P* < 0.05, correlation coefficient > 0.25), and most of them were related to metabolism (Fig. 10). 8 KEGG pathways, such as galactose metabolism, focal adhesion, ERBB signaling pathway and adherens junction, were positively correlated with risk score, and 9 KEGG pathways, such as oxidative phosphorylation, drug metabolism cytochrome p450 and tyrosine metabolism, were negatively correlated with risk score (Fig. 10).

### Comparison with other prognostic models

**Fig. 11:**
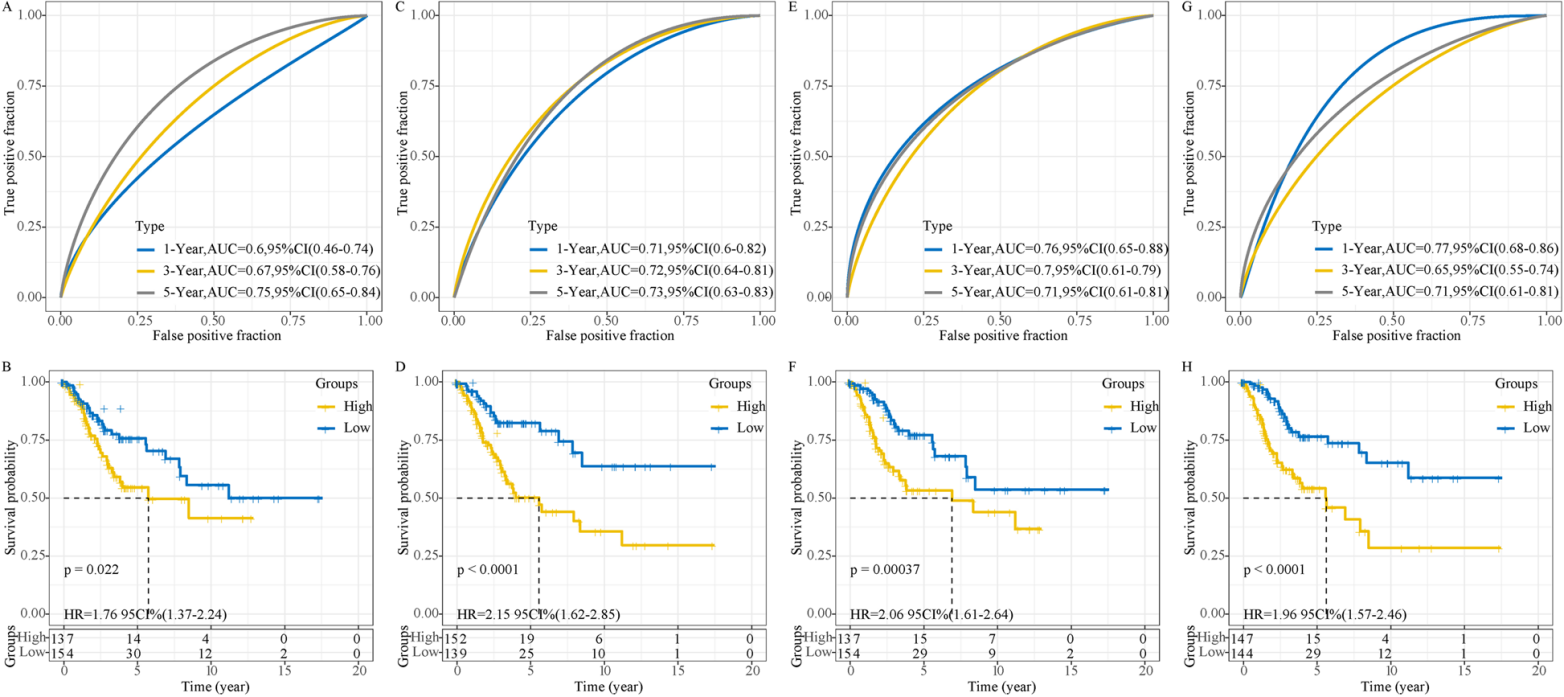
Comparison with other four prognostic models from published literatures. ROC curves and Kaplan–Meier survival curves of 5-gene signature from Ju et al. **A-B**, 5-gene signature from Cai et al. **C-D**, 5-gene signature from Liu et al. **E–F**, and 8-gene signature from Xie et al. **G-H**. HR, hazard ratio. CI, confidence interval

Previous studies have proposed a series of prognostic signatures for cervical cancer, we finally included four other prognostic models that used the same TCGA dataset and whose number of genes were close to our signature. To ensure a comparable standard, the method used in the current research was applied to calculate risk score for each TCGA sample using the four prognostic models. Kaplan–Meier survival curves and ROC curves of four models were plotted (Fig. 11). These four prognostic models could clearly divide samples into high-risk and low risk groups with distinctly different prognosis (*P* < 0.05). Compared with our prognostic signature, a 5-gene signature by Ju et al. had the highest AUC (0.75, 95%CI = 0.65–0.84) of 5-year prognosis (Fig. 11A and B), and an 8-gene signature by Xie et al. had the highest AUC (0.77, 95%AUC = 0.68–0.86) of 1-year prognosis (Fig. 11G and H). However, our 5-gene signature was relatively more accurate in predicting 1-year, 3-year and 5-year prognosis, with an AUC of 0.74, 0.72 and 0.74 respectively (Fig. 5B and C).

## Discussion

In the present study, we proposed a 5-gene prognostic signature based on integrated analysis of CNV data and expression profiles. The 5-gene signature can effectively classify cervical cancer patients into high-risk and low-risk groups with distinctly different prognosis, and it showed a robust performance in both TCGA and GSE44001 datasets. This work was the first attempt to report a prognostic signature based on combined data of CNVs and mRNAs. Differential gene expression and CNV were both present in the 5 prognostic genes, therefore, expression level and CNV detection may both serve as detective means for cervical cancer theoretically. CNVs are more convenient to be detected compared with mRNA expression, therefore, detecting CNVs of the 5 prognostic genes could serve as a preliminary screen of high-risk cervical cancer patients.

Risk score can be calculated according to the expression of 5 prognostic genes (*APOD*, *ARMCX1*, *GALNT3*, *HK2* and *HLF*), which emerged as an independent risk factor in Cox regression analysis. For a convenient use in clinical practice, we constructed a nomogram based on risk score for predicting patients’ survival chance. DCA evaluation showed a better performance of the nomogram than risk score. Thus, the nomogram was recommended as a predictive measurement for prognosis prediction of cervical cancer patients.

We further studied the 5 prognostic genes, and they were found to be associated with cancers reported in the previous research. APOD is one of apolipoproteins (APOs) that bind lipids and transport them to various tissues during lipid metabolism. A series of APOs have been illustrated to be associated with cancer development [28]. For example, APOA family, especially APOA1, has been considered as an independent predictor for progressing cancers such as non-small cell lung adenocarcinoma, ovarian cancer, colorectal cancer and prostate cancer [28]. APOD is associated with high density lipoprotein (HDL), and is identified as a predictive biomarker hepatocellular carcinoma [29]. Low expression level of APOD is predictive of unfavorable prognosis in many cancer types, for instance, colorectal cancer [30], ovarian cancer [31] and breast cancer [32]. However, APOD is high-expressed in other cancers such as melanoma [33] and renal cell cancer [34]. In our study, APOD was lower-expressed in high-risk group as compared with low-risk group, suggesting that low-expressed APOD was associated with unfavorable prognosis of cervical cancer.

ARMCX1 (also known as ALEX1) belongs to ARMCX family clustered in X chromosome, which could regulate protein–protein interaction. Evidence has shown that ARMCX family is involved in tumorgenesis and tumor progression through oncogenic pathways such as WNT signaling pathway [35, 36]. ARMCX1 is identified as a prognostic biomarker in ovarian cancer [37], colorectal cancer [38] and also in cervical cancer [39]. Zeng et al. observed a higher expression of ARMCX1 in cervical cancer tissues than normal cervical tissues [39]. Similarly, our study found that ARMCX1 was high- expressed in high-risk group with poor prognosis.

GALNT3 is an enzyme for *O*-glycosylation, and down-regulation of GALNT3 has been reported to be associated with poor prognosis of lung adenocarcinoma [40], colorectal cancer [41], and pancreatic cancer [42, 43]. Conversely, high expression of GALNT3 is detected in oral squamous cell carcinomas [44] and ovarian cancer [45]. Here in cervical cancer, we observed up-regulated GALNT3 compared with normal cervical tissues, particularly in high-risk group. Wang et al. discovered that knockdown of GALNT3 is correlated with cell adhesion molecules β-catenin and E-cadherin in ovarian cancer, which supported the invasion of epithelial ovarian cancer.

HK2 is a hexokinase with a critical role in glycolysis. Studies have found that HK2 is overexpressed in many cancer types, and that the inhibition of HK2 expression can inhibit cancer cell proliferation [46, 47]. Consistently, in this study, HK2 was also overexpressed in cervical cancer tissues and its high expression was associated with poor OS. Previously, knockdown of HK2 in cervical cancer cells has been observed to inhibit expression of AKT and mTOR, which are involved in cancer progression [48]. Such a finding supported that HK2-related pathway can serve as a potential target for treating cervical cancer. Hepatic leukemia factor (HLF) is considered as an oncogenic transcript factor, and its high expression correlates with favorable prognosis in many cancers such as glioma [49] and non-small cell lung cancer [50]. This study also found that high expression of HLF was associated with a better prognosis of cervical cancer.

Overall, we proposed a 5-gene prognostic signature in which each component gene was closely related to various cancers. In addition, we assessed the TME of high-risk and low-risk groups, and detected a significant difference between them, indicating that these genes may participate in the modulation of immune infiltration. Compared with high-risk group, low-risk group manifested higher immune infiltration, especially activated B cells and CD8 T cells, suggesting that low-risk group had a more activated anti-tumor response and therefore may result in a favorable prognosis. In comparison with other prognostic signatures calculated by the same methodology, our 5-gene signature showed the highest AUC. However, the signature established based on bioinformatics analysis requires further validation in clinical practice.

## Conclusions

In conclusion, this study developed a novel prognostic signature with a robust performance in different datasets. The study provided the first comprehensive assessment of prognostic genes according to the relation between CNVs and cervical cancer. In addition to predictive ability for cervical cancer prognosis, the 5 prognostic genes emerged as new targets for further understanding the mechanisms of cervical cancer development.

## Supplementary Information

**Additional file 1:** **Supplementary Figure S1.** The PCA plot before (A) and after (B) removing batch effects of TCGA and GTEx datasets. PCA, principle component analysis.

**Additional file 2:** **Supplementary Figure S2.** The density plot of *P* values on univariate Cox regression between genes in training and test groups. Log-rank test was performed.

**Additional file 3:** **Supplementary Figure S3.** The intersection between 6608 genes from differential CNVs and 788 DEGs.

**Additional file 4:** **Supplementary Figure S4.** The location of 5 prognostic genes in genome.

**Additional file 5:** **Supplementary Figure S5.** The expression of 5 prognostic genes in normal and cancer samples. EXP, expression.

**Additional file 6:** **Supplementary Figure S6.** Pearson correlation analysis between CNV and the expression of 5 prognostic genes.

**Additional file 7:** **Supplementary Figure S7.** The performance of 5-gene prognostic model in test group. (A) The survival status and expression of 5 genes of each sample ranking by risk score. (B) ROC curve of predicting 1-year, 3-year and 5-year survival. (C) Kaplan-Meier survival curve of high-risk and low-risk groups. Log-rank test was performed. AUC, area under ROC curve. CI, confidence interval. HR, hazard ratio.

**Additional file 8:** **Supplementary Figure S8.** The performance of 5-gene prognostic model in GSE44001 dataset. (A) The survival status and expression of 5 genes of each sample ranking by risk score. (B) ROC curve of predicting 1-year, 3-year and 5-year survival. (C) Kaplan-Meier survival curve of high-risk and low-risk groups. Log-rank test was performed. AUC, area under ROC curve. CI, confidence interval. HR, hazard ratio.

**Additional file 9:** **Supplementary Figure S9.** The distribution of risk score in different clinical features including survival status (A), T stage (B), N stage (C), M stage (D), stage (E), age (F), HPV status (G) and grade (H). ANOVA was performed. *P < 0.05.

**Additional file 10:** **Supplementary Table S1.** The clinical information of TCGA, GTEx and GSE44001 datasets.

**Additional file 11:** **Supplementary Table S2.** The clinical information of training group and test group.

**Additional file 12:** **Supplementary Table S3.** The list of 255 common genes between genes in CNVs and DEGs.

**Additional file 13:** **Supplementary Table S4.** The results of univariate Cox regression analysis for screening prognostic genes significantly associated with prognosis.

## Data Availability

The datasets generated and/or analyzed during the current study are available in the [GSE44001] repository in [https://www.ncbi.nlm.nih.gov/geo/query/acc.cgi?acc=GSE44001].

## Abbreviations

AIC: Akaike information criterion
APOs: Apolipoproteins
AUC: Area under ROC curve
CI: Confidence interval
CNV: Copy number variation
DCA: Decision curve analysis
DEGs: Differentially expressed genes
ESTIMATE: Estimation of STromal and Immune cells in MAlignant Tumours using Expression data
FIGO: The International Federation of Gynecology and Obstetrics
GEO: Gene Expression Omnibus
GO: Gene ontology
HLF: Hepatic leukemia factor
HPV: Human papillomavirus
HR: Hazard ratio
KEGG: Kyoto Encyclopedia of Genes and Genomes
MCP-counter: Microenvironment Cell Populations-counter
MDSC: Myeloid-derived suppressor cells
OS: Overall survival
PD-1: Programmed death receptor-1
ROC: Receiver operating characteristic
ssGSEA: Single sample gene set enrichment analysis
TCGA: The Cancer Genome Atlas
TME: Tumor microenvironment
