# Supplementary figures and images for: Developing a 5-gene prognostic signature for cervical cancer by integrating mRNA and copy number variations
Source: BMC Cancer. 2022 Feb 21;22:192. doi: 10.1186/s12885-022-09291-z (PMC8859909; doi:10.1186/s12885-022-09291-z)

A

Before

●

GTEx

●

TCGA

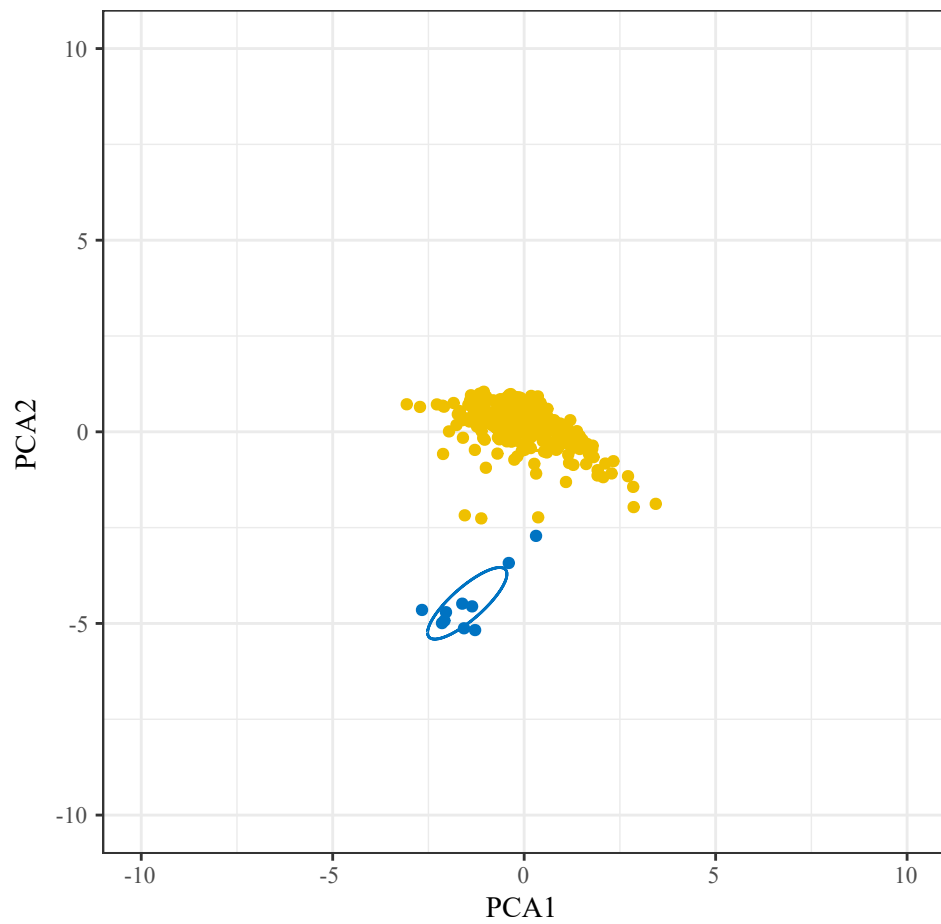

B

After

●

GTEx

●

TCGA

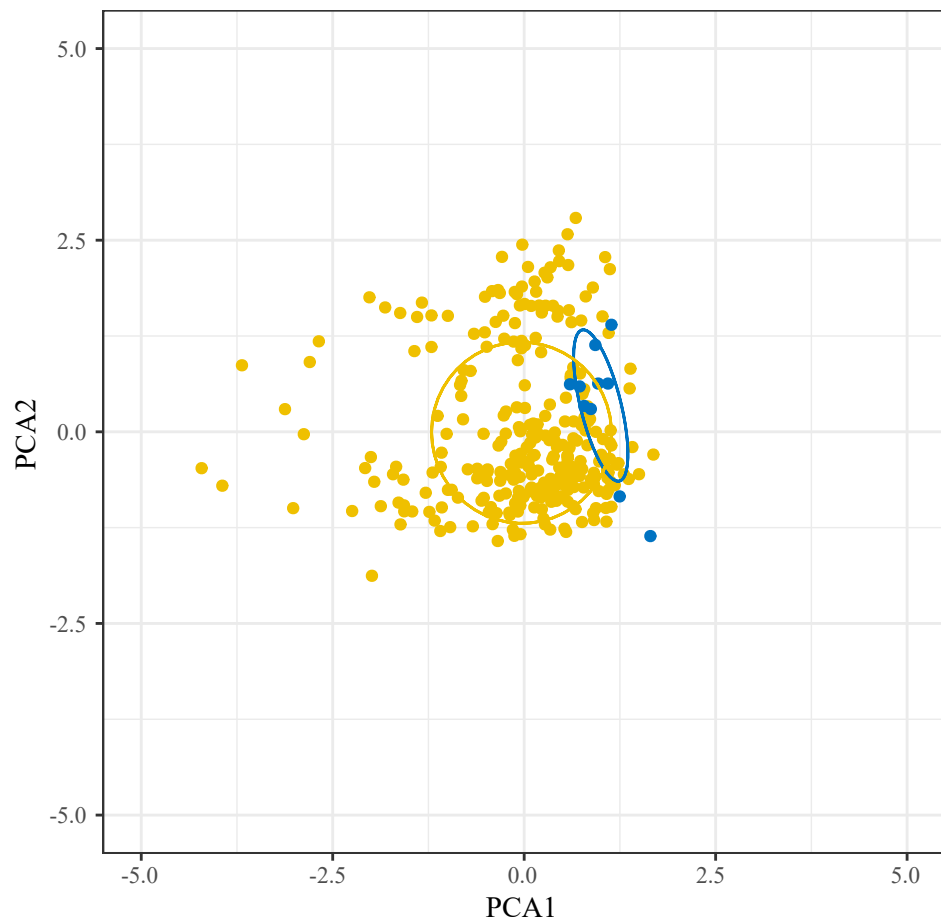

Supplement: Supplementary file 1 — Additional file 1: Supplementary Figure S1. The PCA plot before (A) and after (B) removing batch effects of TCGA and GTEx datasets. PCA, principle component analysis. [file 12885_2022_9291_MOESM1_ESM.pdf]

Data  Test  Train

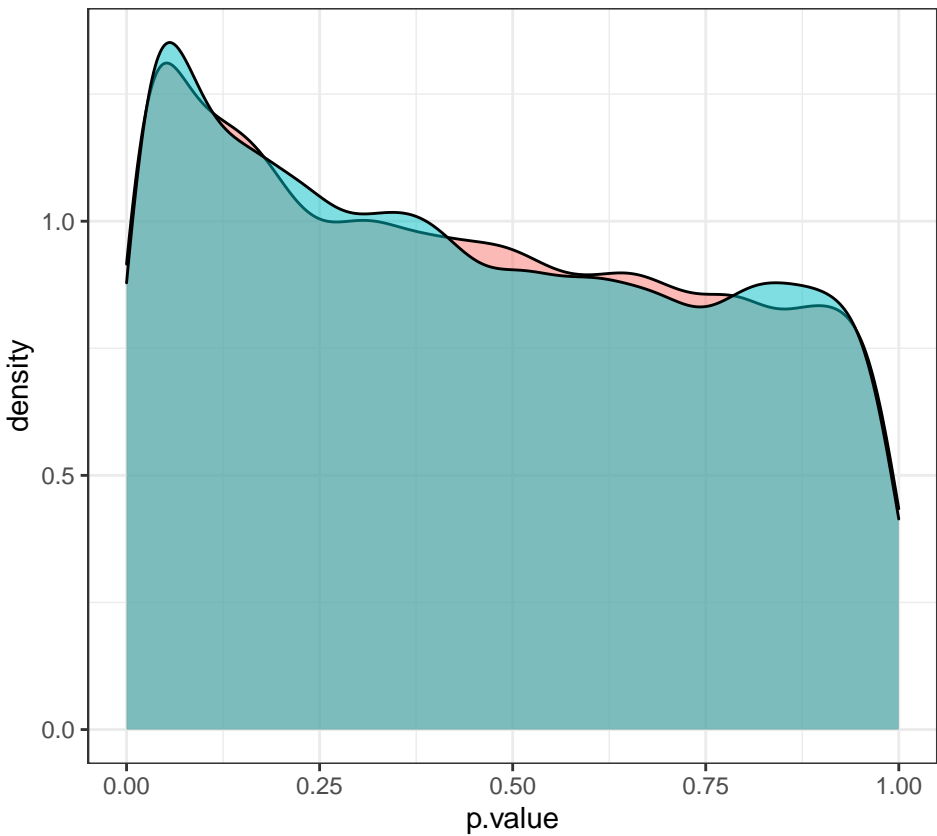

Supplement: Supplementary file 2 — Additional file 2: Supplementary Figure S2. The density plot of P values on univariate Cox regression between genes in training and test groups. Log-rank test was performed. [file 12885_2022_9291_MOESM2_ESM.pdf]

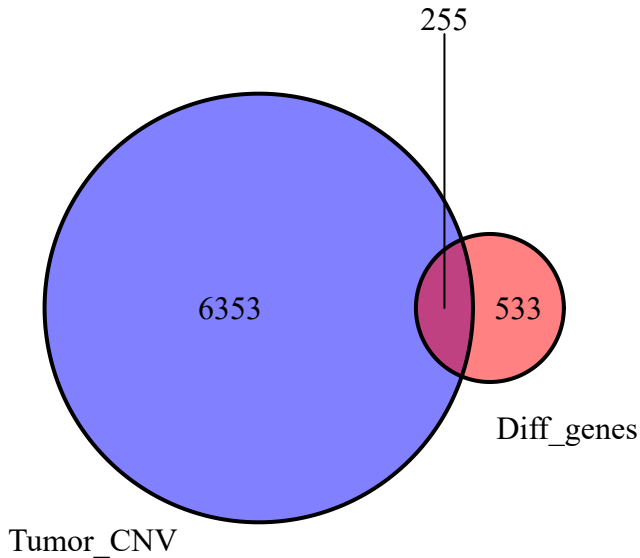

Supplement: Supplementary file 3 — Additional file 3: Supplementary Figure S3. The intersection between 6608 genes from differential CNVs and 788 DEGs. [file 12885_2022_9291_MOESM3_ESM.pdf]

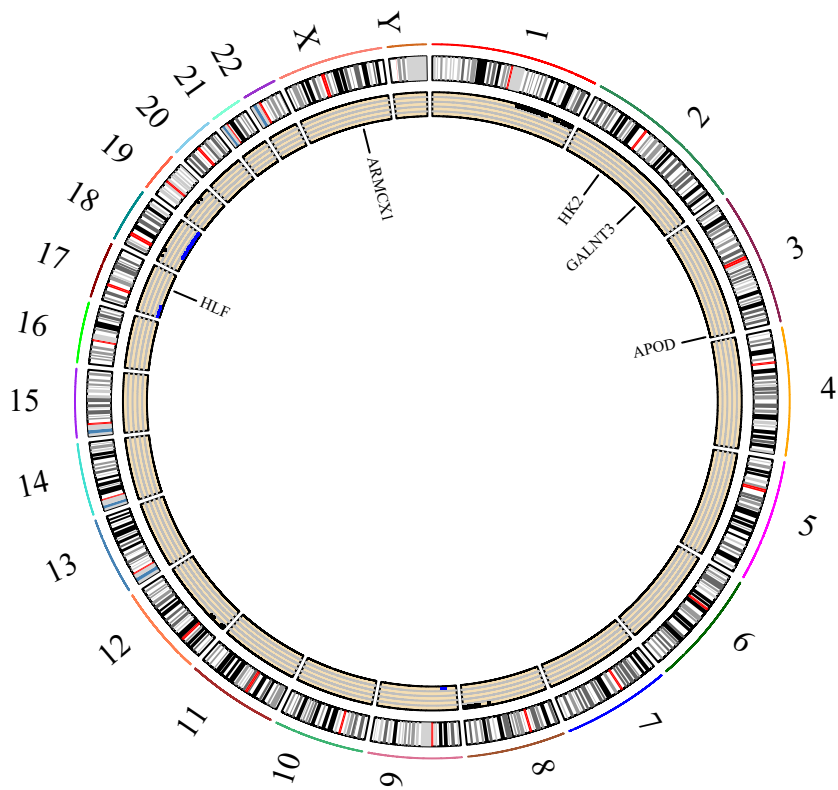

Supplement: Supplementary file 4 — Additional file 4: Supplementary Figure S4. The location of 5 prognostic genes in genome. [file 12885_2022_9291_MOESM4_ESM.pdf]

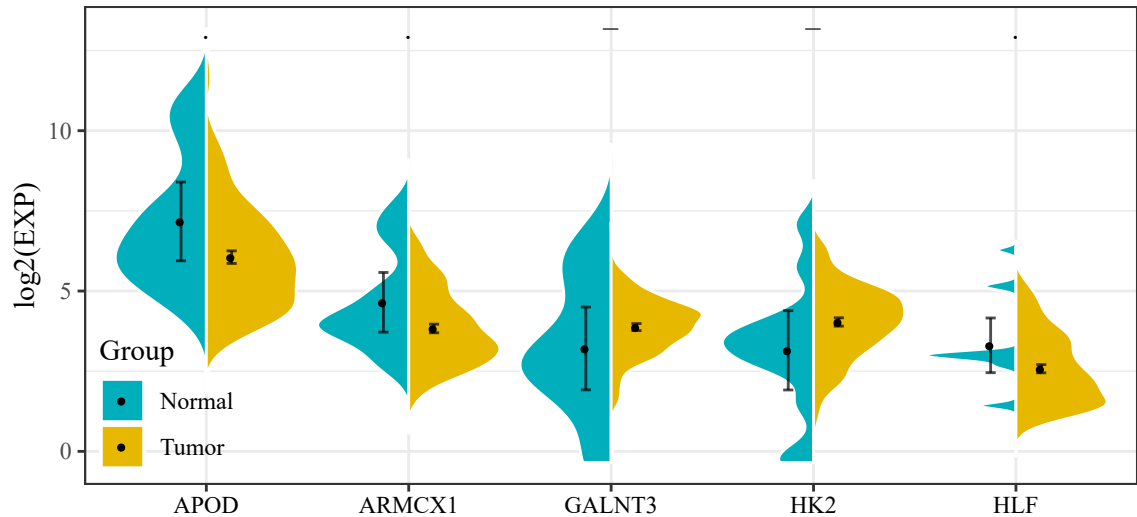

Supplement: Supplementary file 5 — Additional file 5: Supplementary Figure S5. The expression of 5 prognostic genes in normal and cancer samples. EXP, expression. [file 12885_2022_9291_MOESM5_ESM.pdf]

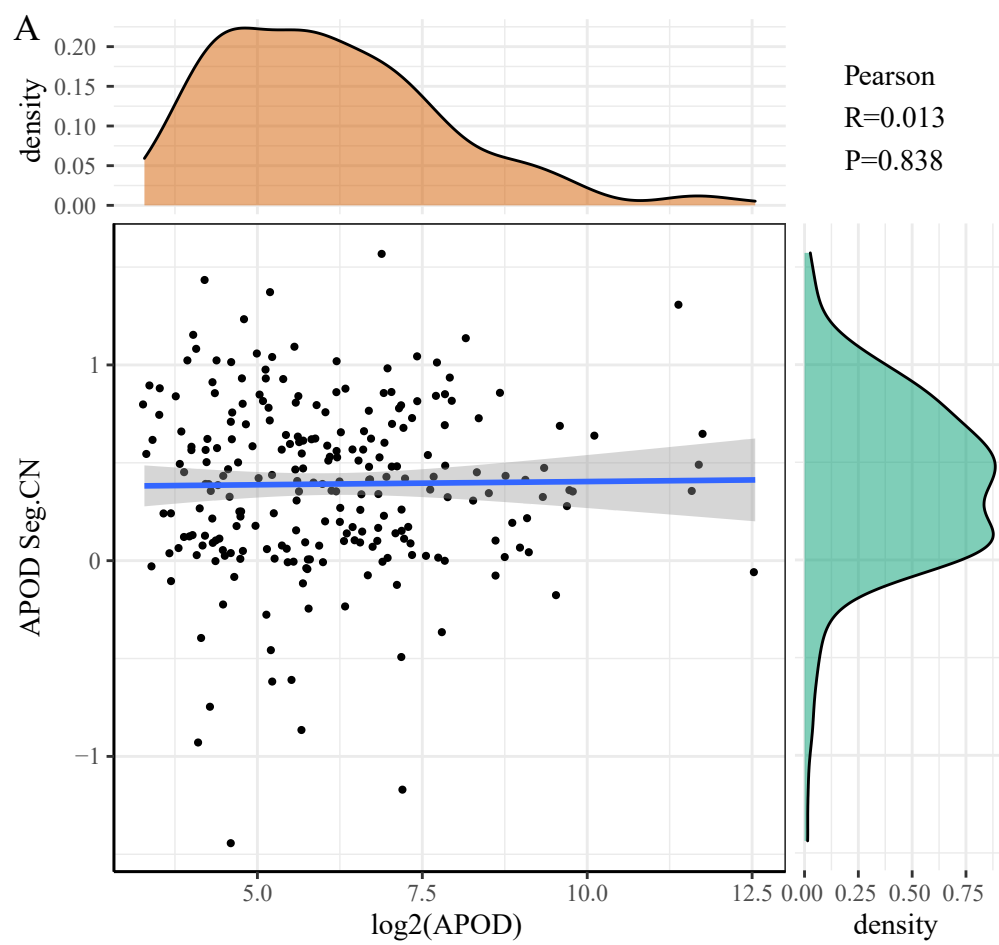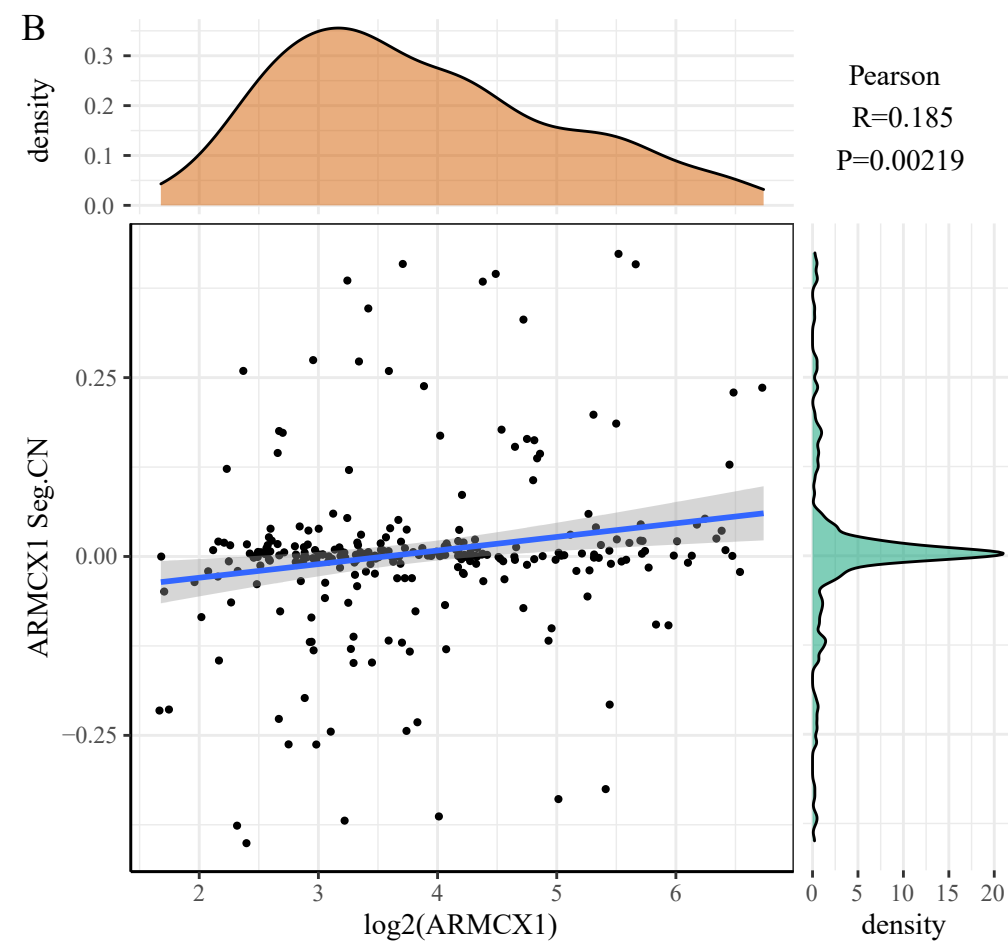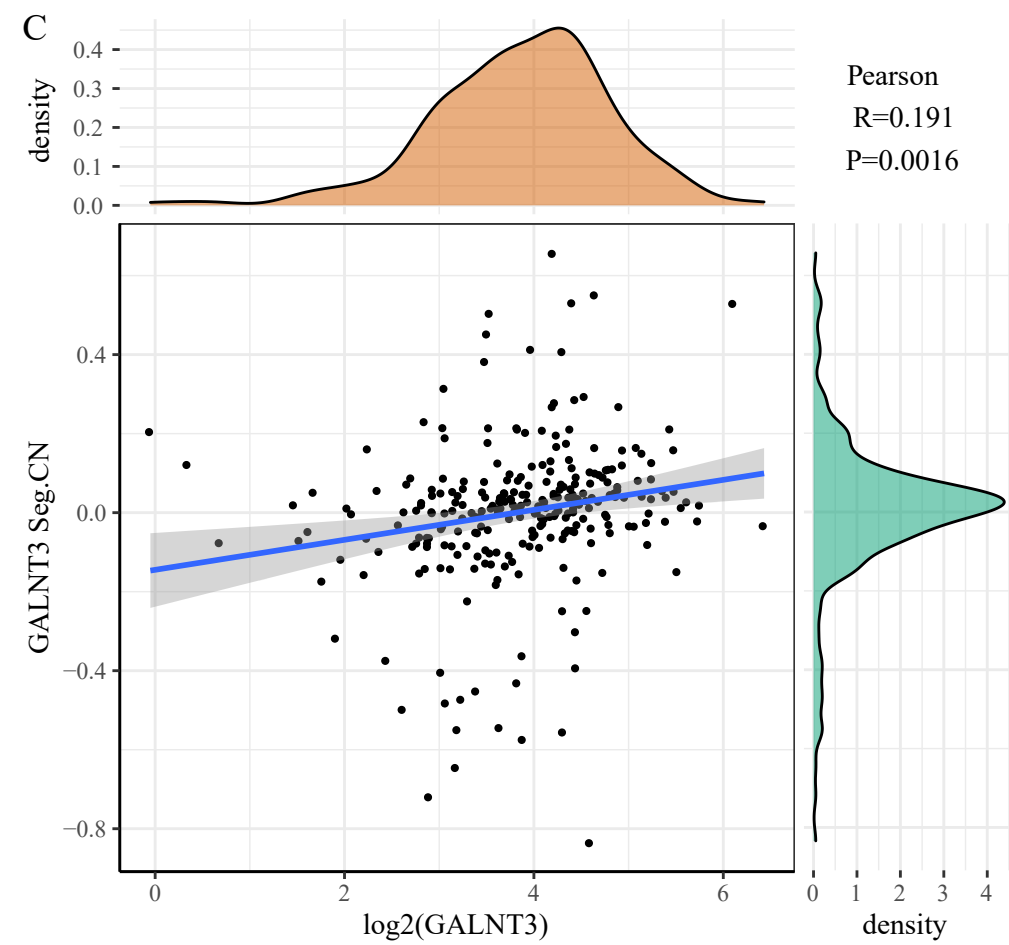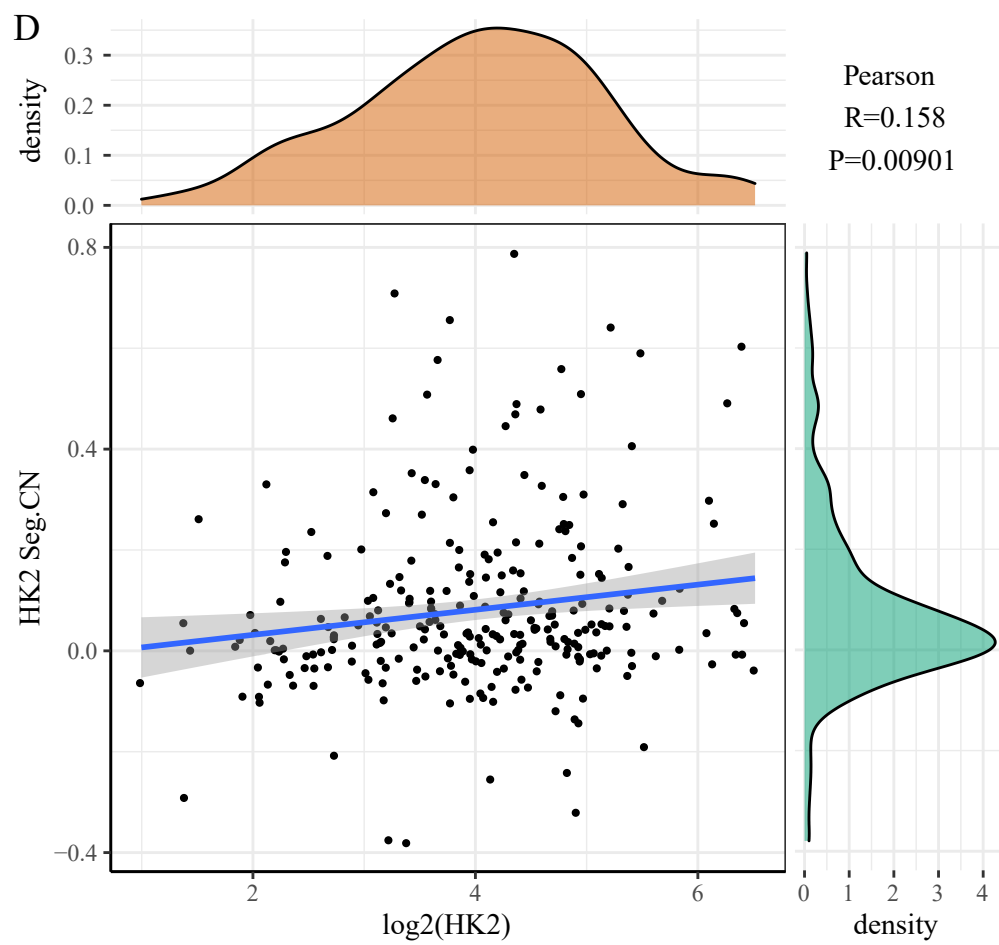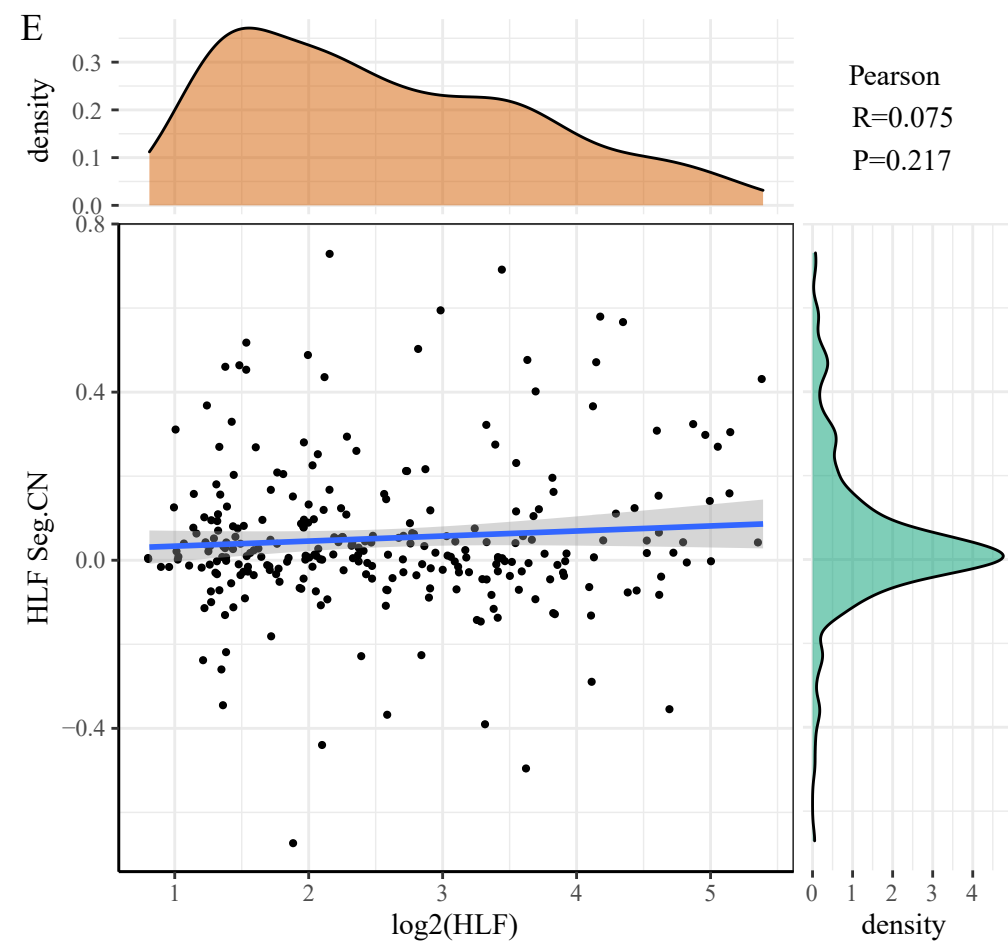

Supplement: Supplementary file 6 — Additional file 6: Supplementary Figure S6. Pearson correlation analysis between CNV and the expression of 5 prognostic genes. [file 12885_2022_9291_MOESM6_ESM.pdf]

A

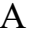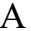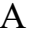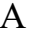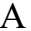

A

A

A

Supplement: Supplementary file 7 — Additional file 7: Supplementary Figure S7. The performance of 5-gene prognostic model in test group. (A) The survival status and expression of 5 genes of each sample ranking by risk score. (B) ROC curve of predicting 1-year, 3-year and 5-year survival. (C) Kaplan-Meier survival curve of high-risk and low-risk groups. Log-rank test was performed. AUC, area under ROC curve. CI, confidence interval. HR, hazard ratio. [file 12885_2022_9291_MOESM7_ESM.pdf]

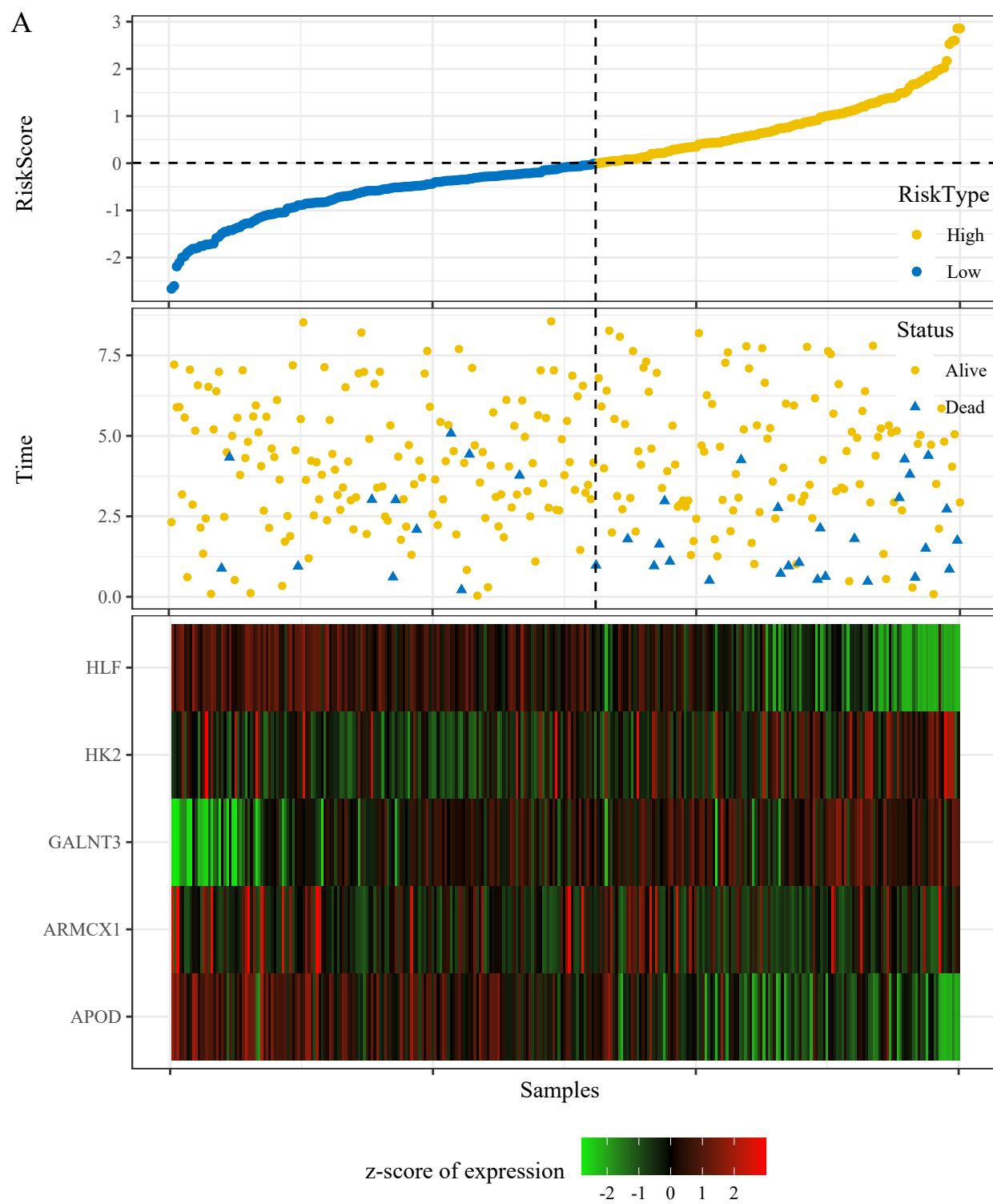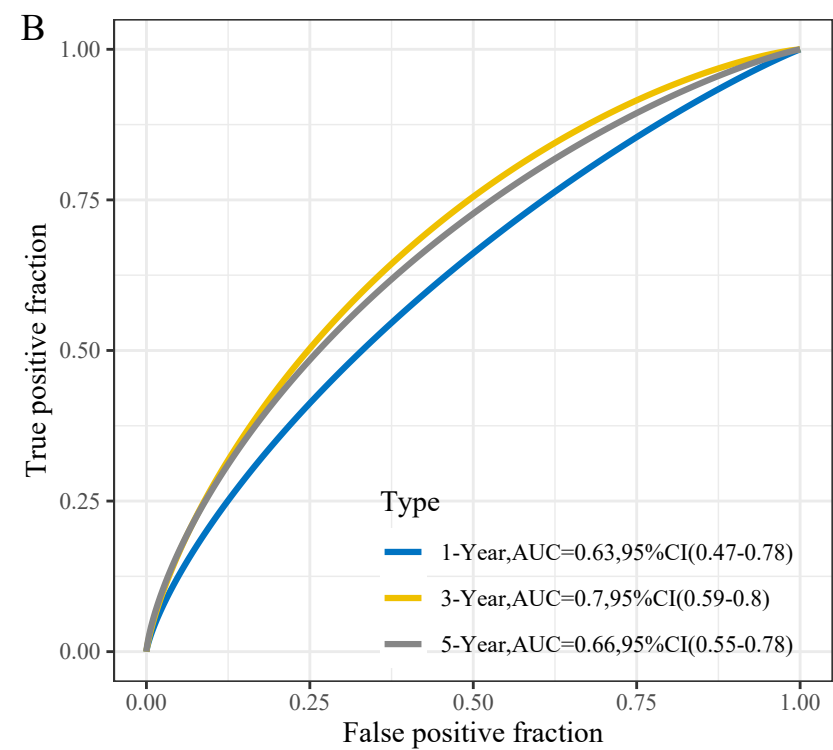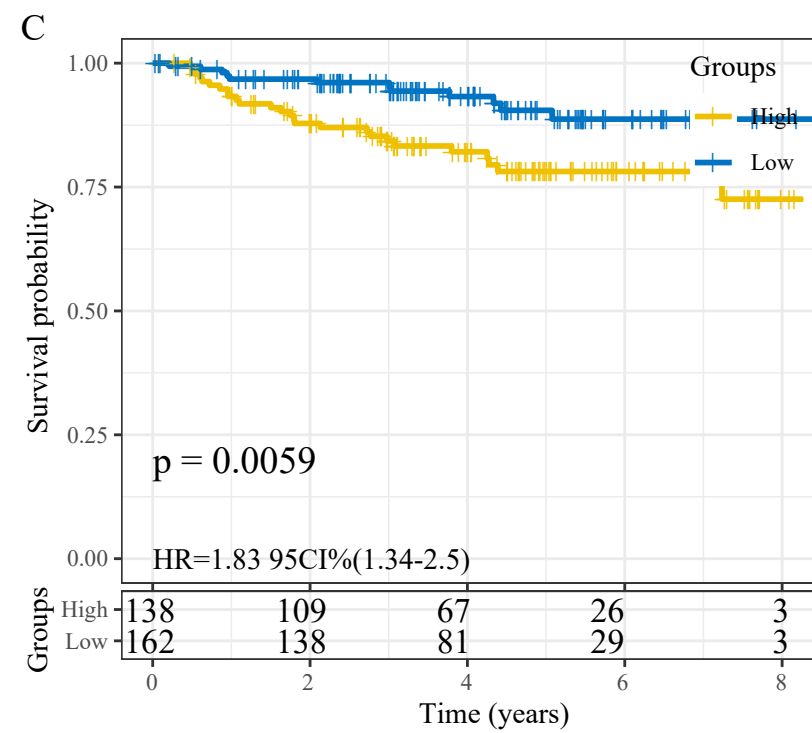

Supplement: Supplementary file 8 — Additional file 8: Supplementary Figure S8. The performance of 5-gene prognostic model in GSE44001 dataset. (A) The survival status and expression of 5 genes of each sample ranking by risk score. (B) ROC curve of predicting 1-year, 3-year and 5-year survival. (C) Kaplan-Meier survival curve of high-risk and low-risk groups. Log-rank test was performed. AUC, area under ROC curve. CI, confidence interval. HR, hazard ratio. [file 12885_2022_9291_MOESM8_ESM.pdf]

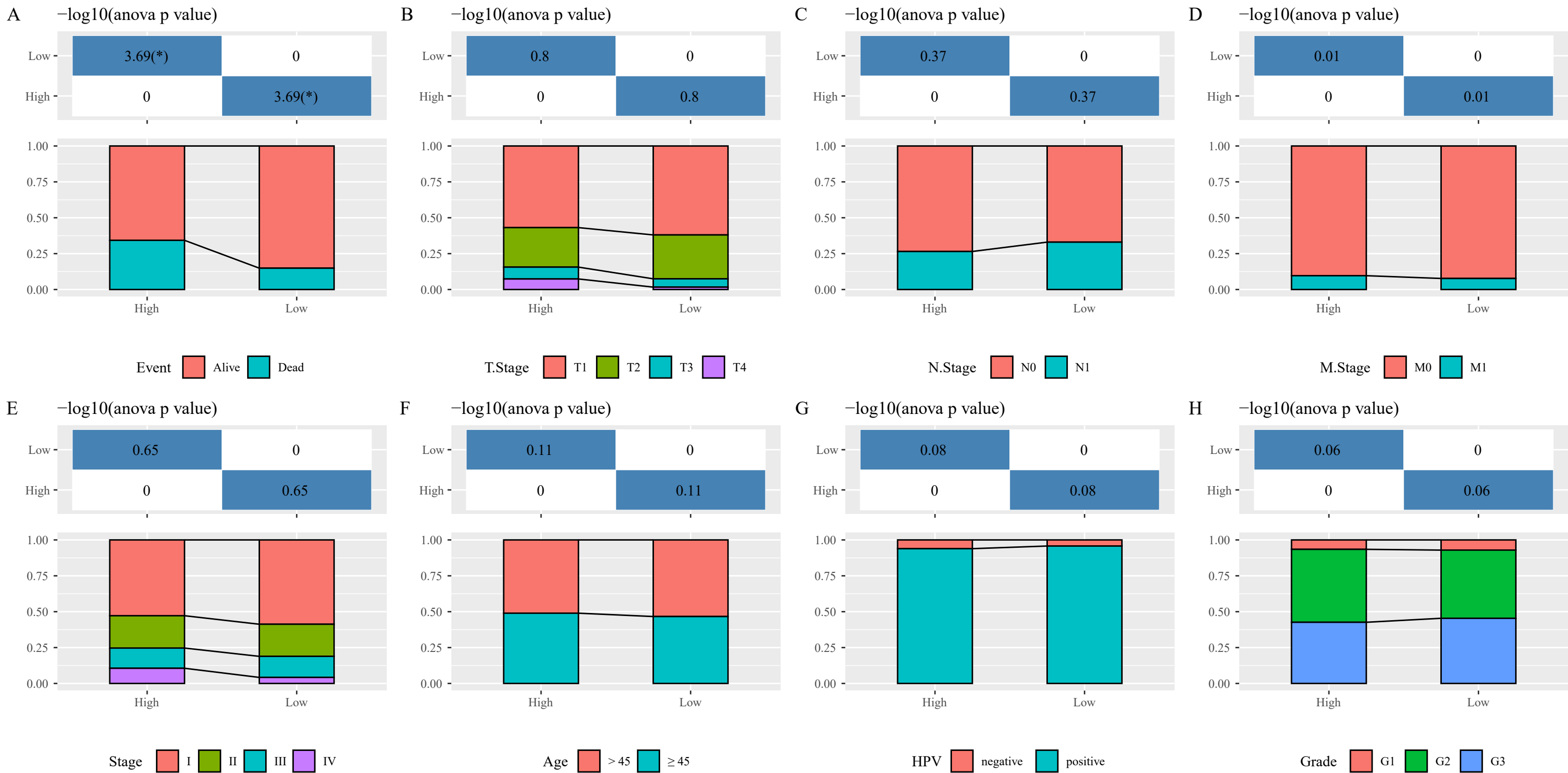

Supplement: Supplementary file 9 — Additional file 9: Supplementary Figure S9. The distribution of risk score in different clinical features including survival status (A), T stage (B), N stage (C), M stage (D), stage (E), age (F), HPV status (G) and grade (H). ANOVA was performed. *P < 0.05. [file 12885_2022_9291_MOESM9_ESM.pdf]
